# A mark–recapture approach for estimating population size of the endangered ringed seal (*Phoca hispida saimensis*)

**DOI:** 10.1371/journal.pone.0214269

**Published:** 2019-03-22

**Authors:** Meeri Koivuniemi, Mika Kurkilahti, Marja Niemi, Miina Auttila, Mervi Kunnasranta

**Affiliations:** 1 Department of Environmental and Biological Sciences, University of Eastern Finland, Joensuu, Finland; 2 Natural Resources Institute Finland, Turku, Finland; 3 Metsähallitus, Parks & Wildlife Finland, Savonlinna, Finland; 4 Natural Resources Institute Finland, Joensuu, Finland; University of Sydney, AUSTRALIA

## Abstract

Reliable population estimates are fundamental to the conservation of endangered species. We evaluate here the use of photo-identification (photo-ID) and mark-recapture techniques for estimating the population size of the endangered Saimaa ringed seal (*Phoca hispida saimensis)*. Photo-ID data based on the unique pelage patterns of individuals were collected by means of camera traps and boat-based surveys during the molting season in two of the species’ main breeding areas, over a period of five years in the Pihlajavesi basin and eight years in the Haukivesi basin. An open model approach provided minimum population estimates for these two basins. The results indicated high survival rates and site fidelity among the adult seals. More accurate estimates can be obtained in the future by increasing the surveying effort both spatially and temporally. The method presented here proved effective for evaluating population size objectively, whereas the results of the current snow lair censuses are dependent on varying winter conditions, for instance. We therefore suggest that a photo-ID-based non-invasive mark-recapture method should be used for estimating Saimaa ringed seal abundances in order to ensure reliable, transparent population monitoring under changing climatic conditions.

## Introduction

Reliable scientific estimates of abundance, distribution, and density are essential for the successful management and conservation of any animal species, and in the case of endangered populations, an accurate knowledge of trends in abundance can allow the development of effective conservation measures. It is not always easy to estimate population size, however, especially for rare and elusive species living in harsh environments. In the case of pinnipeds, traditional census methods are generally based on aerial surveys carried out during the molting or breeding season (e.g., [1–4]), when the seals spend most of their time out of the water, on land or ice platforms. The ringed seal (*Phoca hispida*) is one of the most dependent of all the pinnipeds on ice and snow for its breeding, resting, and molting. In addition, estimates of its abundance are dependent on the ice platform, because censuses are typically carried out during the ice-cover season, in early spring. This means that the mild winters brought about by climate change may not only hamper the ringed seals’ breeding success [5], but may also affect the reliability of monitoring their populations [6].

The main threats to the small endemic population of the endangered Saimaa ringed seal (*P*.*h*. *saimensis*) in Lake Saimaa, are the negative impacts of climate change, e.g. reduction of the number of available breeding habitats due to a decline in the ice cover and protective snow cover [7], which will thus reduce pup survival [8] together with high by-catch mortality [9]. These seals live in a freshwater lake habitat and exhibit a high degree of site fidelity [10–12]. The latest genetic results point to extremely low genetic diversity and a division of the population into small, semi-isolated subpopulations inhabiting different parts of the lake [13]. Population estimates in the past have been highly uncertain, and both the methods and their outcomes have varied greatly [14]. Kokko et al. [15] used bounty statistics to estimate the abundance of this subspecies historically, resulting in estimates of between 100 and 1,300 individuals for the year 1900, while even higher estimates of 2,000–4,000 seals have been proposed for 5,000 years ago [16]. The decline in population size reached its turning point (at around 130–160 individuals) in the mid-1980´s [17]. Currently the population is around 400 individuals and according to the past years population estimates made by the national authorities Metsähallitus [18] the population is known to be growing slowly [18].

Traditional transect-based aerial surveys of ringed seals during the peak of the molting season on the ice [19–21] are not used for population estimation in Lake Saimaa due to the mosaic lake environment and the individuals’ preference for molting later in the spring on a terrestrial platform. The current method is thus based on the numbers of snow lairs observed annually during the late nursing season in April. The females give birth to a single pup in subnivean lairs constructed in snowdrifts along the shores of islands or islets in February-March. In addition, males and non-breeding females use snow lairs for hauling out [22]. The census approach takes these lair types (birth or haul-out lairs) into account, the locations of the lairs, and the numbers of pups born (see [23]). However, not all the technical aspects of the method used at present for estimating the population size of the Saimaa ringed seal have been published in detail, but it relies largely on expert opinion. Although censuses have been carried out relatively systemically since the 1980’s, the counting of subnivean snow structures has become a more challenging task during the last decade due to poor snow and ice conditions [8]. Consequently, the estimating of both the abundance of these seals and their distribution will become more difficult under changing climatic conditions in the future, whereas reliable data are urgently needed for conservation purposes. In addition, there has been distrust among many local residents and some interest groups with respect to the current official population estimates [24]. This highlights the need to develop a systematic approach to the estimation of population size with acceptable levels of precision and accuracy, as is essential for conservation purposes and for the social acceptability of the resulting conservation measures.

Photo-identification (photo-ID)-based mark-recapture studies of estimating population have increasingly been used in studies of marine mammals, mostly cetaceans (e.g., [25–30]), and these techniques has also been utilized recently for estimating pinniped populations [31–35]. Since the permanent pelage pattern of ringed seals is individual and lifelong, photo-ID has been shown to be a suitable approach for monitoring the Saimaa ringed seal [36]. The goal of this study was to establish a photo-ID-based mark-recapture tool for monitoring Saimaa ringed seals and to develop and recommend a new approach for monitoring this endangered population in the future. The specific aims were 1) to estimate the size of the population in two main breeding areas using camera trapping and boat-based surveys, and 2) to compare estimates obtained with data of different types (camera trap data and boat-based surveys) in order to validate the most reliable and effective sampling procedure.

## Materials and methods

### Photo-identification data

**Fig 1:**
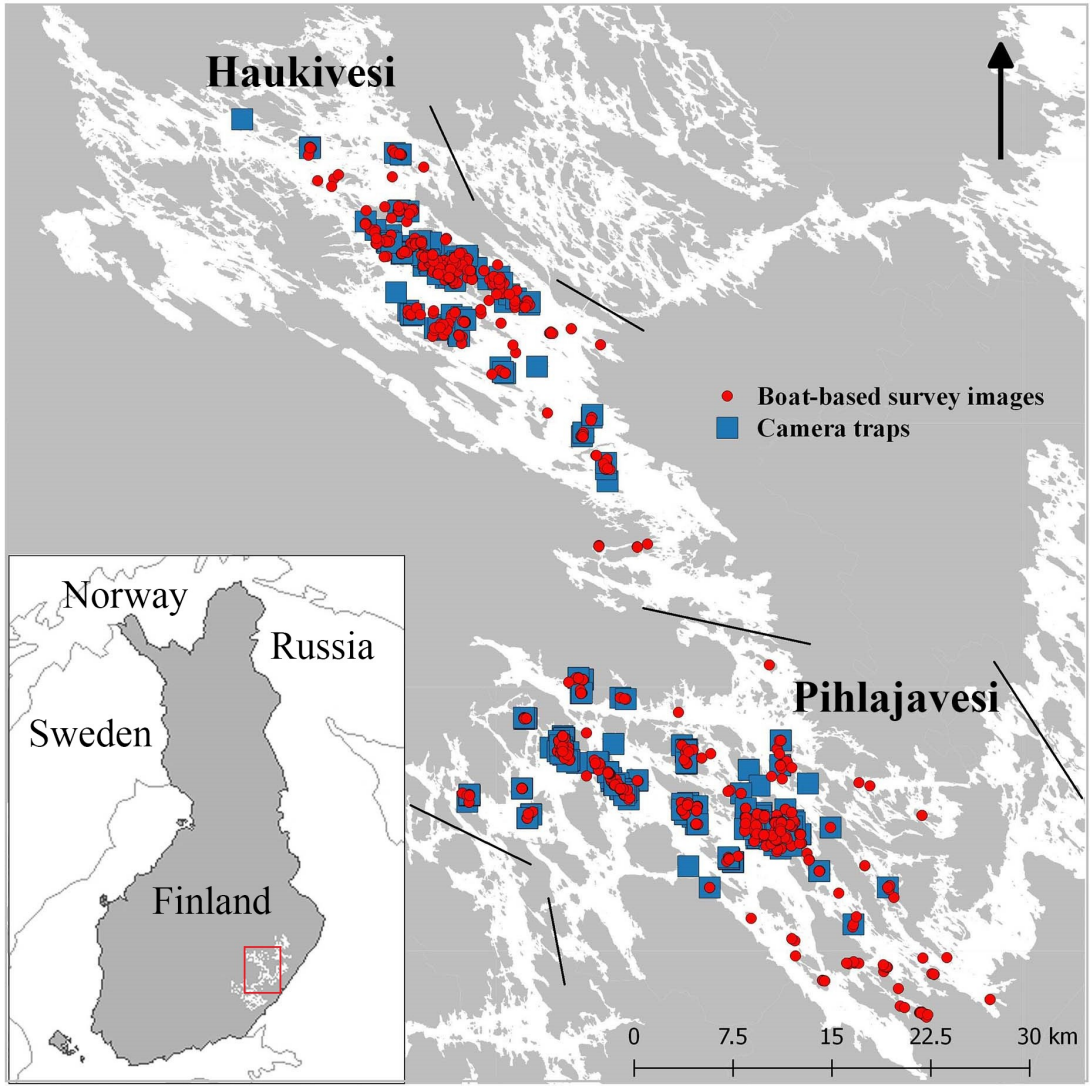
Photo-ID study locations in Lake Saimaa. Camera trap sites (blue box) and locations where ringed seals were photographed during the boat-based surveys (red dots) in the Pihlajavesi and Haukivesi basins. Basemap (C) Land Survey of Finland 4/2018.

The research was carried out in Lake Saimaa, Finland’s largest lake (62°13’–61°34’N, 28°08’–29°06’E, Fig 1), the data being collected in two of the main breeding areas of the Saimaa ringed seal in five and eight consecutive years, 2013–2017 in the Pihlajavesi and 2010–2017 in the Haukivesi basin. These two bodies of water account for approximately one fourth of the total lake area (4,400 km^2^) [37] and possess around 55% of the ringed seal population of the whole of Lake Saimaa as estimated in recent years [18]. In the early years the areas surveyed amounted to about half of each lake basin, Pihlajavesi and Haukivesi, and included the most intensively used molting sites, but since 2016 the survey has been extended to include approximately 85% of the area of these basins.

The photo-ID data were collected during the annual molting season in May-June, which is the only time of the year when the ringed seals are visible, spending extended periods at terrestrial haul-out sites, typically on the rocky shores of islands or islets and occupied by single individuals or a group of up to five. The fur patterns of the Saimaa ringed seal are clearly visible even at the peak of the molting season. The pups shed their long, grey lanugo hair in lairs, in late April, and after that they are individually identifiable. Due to the new hair, however, the pups do not regularly haul out during their first spring, and therefore they are not included in the present data.

**Fig 2:**
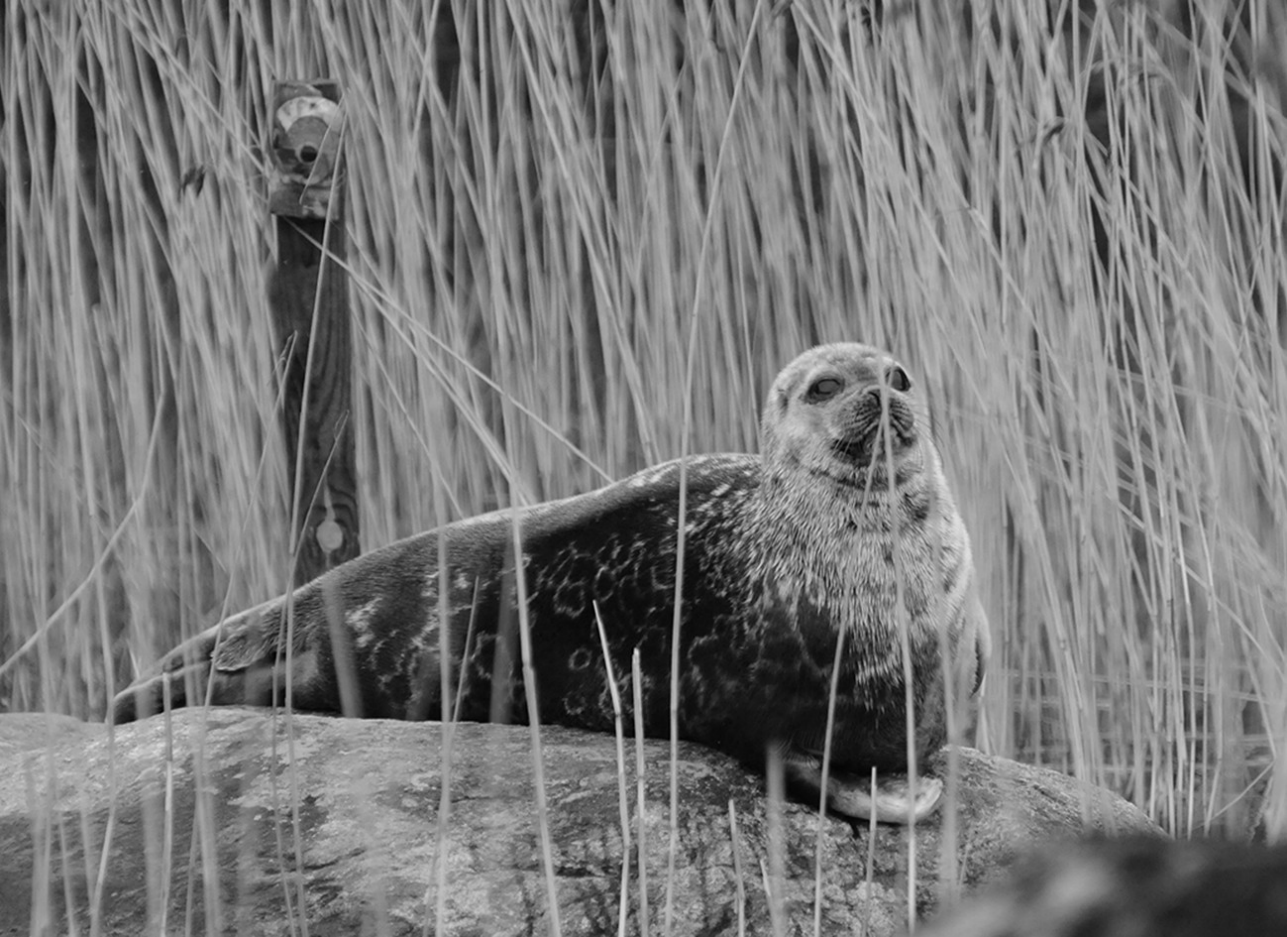
A camera trap and a Saimaa ringed seal at a haul-out site.

The camera trap images were collected using motion-sensitive game cameras (Scout Guard 550VB and 560K-8) and the images from the boat-based surveys were collected using digital single-lens reflex (DSLR) cameras (Nikon D300) equipped with zoom lenses of up to 300 mm. The camera traps were placed on wooden sticks or trees next to the haul-out sites (0.5-2m distance) (Fig 2). The locations of the traps were chosen based on annual observation of hauled out seals and the traps were always set up when no seals were present, however. The cameras were fitted with passive infrared sensors and were set to take photos over a 0.5-2min time span, two images at a time. The memory cards (2–4 GB) were changed 1–3 times a week. The boat-based surveys were performed using 6–8 m powered boats (1–3 in use during each molting season) with 20-60hp outboard engines and either a center or side console and with one or two observers on board. When a seal was sighted, it was approached and passed at reduced speed without any sudden changes in direction and photographed from a maximum distance of 150 m. Notes were made of the GPS location, observation time and number of seals. Surveys were continued throughout the molting season (weather permitting) and attempts were made to cover the whole designated area in each water basin several times during the field season. The camera trapping and boat-based survey were conducted for the same five–year period in the Pihlajavesi basin, but in the Haukivesi basin the camera trapping took place only in the first three years while the boat-based survey continued for eight years in all.

Due to the bilateral asymmetry of the fur patterns on the flanks of the seals, images of the right side of each individual were used as a base for our mark-recapture database. These images were then evaluated for quality in three categories: 1) poor quality or unclear pattern; disqualified, 2) part of the pattern visible but some uncertainty; not used in this work, and 3) at least 50% of the flank visible and at least three clear sections of the pattern shown; approved for the analysis. The sex was determined from the images when the belly was visible [36]. The individuals were identified manually by experienced observers on the basis of their natural ring-shaped fur patterns. The approved re-capture history data were fed into a DISCOVERY data management system [38] and an open web catalogue based on Wildbook (www.norppagalleria.fi). The research was conducted under permits ESAELY/1290/2015, KASELY/2014/2015, POKELY/1232/2015 and POSELY/313/07.01/2012 obtained from the local environmental authorities (ELY-centers) and research permit 921/662/2006 from the government-owned enterprise Metsähallitus.

### Mark-recapture analysis

A total of 6 dataset combinations of re-capture histories (see S1 Table) were analyzed, representing the two water basins (Pihlajavesi, Haukivesi), concentrating on the right side of the seals and employing three image acquisition methods (camera trap, boat-based survey, a combination of these, to achieve the highest number of sightings). Only data obtained during the study seasons (annual molting time) and applying to seals over one-year-old were included in the analysis.

Open population models can be conducted over long periods and operate on the assumption that the population is a non-constant. Thus the POPAN formulation of the Jolly-Seber open population model [39, 40] was chosen here, since 1) the main focus was on the estimation of population size, 2) the areas studied accounted for only one fourth of Lake Saimaa, and 3) the time scale of up to eight years allowed immigration and birth or emigration and death to occur. The assumptions made in this model are: 1) all individuals have an equal capture (re-sight) probability, 2) all individuals have the same survival probability, 3) the markings are permanent and read correctly, 4) all samples are instantaneous, and the animals are released immediately after sampling, and 5) the areas concerned remain constant [25, 41].

For *K* capture occasions, the POPAN model provides *K*—1 estimates of survival (Φ, *Phi*), *K* estimates of capture probability (*p*), *K*– 1 estimates of the entrance probability into the population per occasion (*β*, *pent*) and an estimate of the super-population size (*N*), which is the number of all animals ever present in the population concerned during the study period. This leads to eight possible models and, given *K* occasions (years), to 3*K*-1 parameters in the model (for details, see S2 Table). The simplest model, *Phi*(·), *p*(·), *pent*(·), *N*, consisted of a constant (marked as a dot) survival, a constant capture probability, a constant entrance probability, and super-population size over time, while the most complex model (*Phi*(*t*), *p*(*t*), *pent*(*t*), *N)* implied temporal variations (marked as *t*) in survival, capture probability, and entrance probability. From the estimates of *Phi*, *p*, *pent* and *N* additional derived parameters of yearly population size estimates can be calculated.

However, not all the parameters are cleanly estimable in time-dependent models. Only 3K-1-5 parameters are cleanly estimable (*Phi*_1_ –*Phi*_K-1_, *p*_2_– *p*_*K*-1_, *pent*_2_ –*pent*_*K-1*_*)* while the rest are confounded [39]. With three years’ data this amounts to four parameters, with five years’ data nine parameters and with eight years a total of 18 parameters. The same also applies to the derived parameters, among which the first and last of the yearly population size estimates may be confounded. For three years’ data this means that only the second year is cleanly estimable.

We fitted all eight models to each of the capture history datasets (S2 Table), checking all the parameters carefully as to whether they were properly estimated. If any non-confounded real parameter hit the boundary (0 or 1) and/or a beta parameter standard error was zero or very large (10–100 times larger than the beta parameter estimate), the candidate model was deemed non-acceptable.

Model averaging across the accepted candidate model by AICc weight (or QAICc weight) was used to calculate the weighted averages of the estimated real parameters and derived yearly population estimates [42–45]. All the analyses were conducted using the MARK program with the RMark interface package RMark [43, 45–48]. The goodness-of-fit (GOF) was tested with the U-CARE program [49] and extra binomial variation with the Median C-hat or Bootstrap GOF test (all implemented in MARK).

## Results

An average of 54 game cameras were deployed annually in the vicinity of haul-out sites, and most of them (67%) recorded seals. The durations of the data collection periods varied between 33 and 61 days annually and the number of trapping days from 966 to 1,908. Thus the total camera trapping effort during the eight years in question was 11,050 days (Table 1). Correspondingly, the duration of the boat-based survey varied between 5 and 36 days annually. Altogether around 474,000 digital images of seals were obtained using the game cameras and almost 59,000 images of seals were collected from the boat-based surveys using DSLR cameras.

**Table 1:** Annual Saimaa ringed seal photo-ID data collection efforts (in days) in the Pihlajavesi (PV) and Haukivesi (HV) basins.

Year	Camera trapping period		Total camera trapping days		Boat-based survey	
	PV	HV	PV	HV	PV	HV
**2010**		49		1176		11
**2011**		40		966		12
**2012**		46		967		15
**2013**	36		1184		21	8
**2014**	56		1908		21	5
**2015**	61		1367		28	16
**2016**	50		1596		36	23
**2017**	33		1887		20	17

The highest numbers of identifications in the Pihlajavesi basin (N = 115) were provided by the set of combined camera trap and boat-based survey images, namely 8–25% more than with either method used separately (Table 2). While in the Haukivesi basin it was the boat-based survey that identified the highest number of individuals (N = 68), as it was used for five seasons longer than the camera trapping method. The re-sighting percentages of the individuals observed at least twice varied in the range 53–74% in both water basins (Table 2 and original dataset S1 Table). The sex composition in the combined data for both water basins was 38% females, 28% males and 34% unknown.

**Table 2:** Re-sightings of the Saimaa ringed seals identified in the Pihlajavesi (2013–2017) and Haukivesi basins (2010–2017).

		Pihlajavesi			Haukivesi		
		Camera trap	Boat-based survey	Combined	Camera trap	Boat-based survey	Combined
Observed no. of animals		92	107	115	44	68	51
Observed % of animals							
	Once	45.7	47.7	36.5	47.7	26.5	37.3
	Twice	25.0	23.4	24.3	34.1	16.2	43.1
	Three times	18.5	15.0	13.9	18.2	26.5	19.6
	Four times	6.5	8.4	13.0		11.8	
	Five times	4.3	5.6	12.2		7.4	
	Six times					7.4	
	Seven times					1.5	
	Eight times					2.9	
At least (%)							
	Twice	54.3	52.3	63.5	52.3	73.5	62.7
	Three times	29.3	29.0	39.1		57.4	
	Four times	10.9	14.0	25.2		30.9	
	Five times					19.1	
	Six times					11.8	
	Seventimes					4.4	

Only 18.4% of the re-capture combinations were found in the Haukivesi boat-based surveys (8 years), which provided a very sparse dataset, i.e. only 47 out of a possible 255 (2^8^−1). The other datasets for Pihlajavesi and Haukivesi yielded from 80 to 100% of all the possible re-capture combinations (S1 Table).

Goodness-of-fit tests (GOF) TEST3 (TEST3.SR and TEST3.SM together) conducted with the U-CARE program targets transient animals that are “visiting” in established population [50]. This can be a spatial phenomenon (i.e. individuals moving from one area to another or sub-adult animals leaving for new areas) or temporal (i.e. some of the animals have died after the first re-sight). Only TEST3.SR (standardized Log-Odds-Ratio statistic for transience) was applicable to the three-year data (Lake Haukivesi camera trap and Haukivesi combined), but this did not show any statistically significant transient effect (camera trap p = 0.8902, combined p = 0.64284), which is the expected result when only 3-year datasets were available. GOF TEST3 was conducted for all the Pihlajavesi datasets and the eight-year Haukivesi boat-based survey dataset, and the results indicated a statistically significant transient effect in all the Pihlajavesi datasets (p-values from 0.0009 to 0.027) but not in the Haukivesi boat-based survey dataset (p = 0.1941). Movements of individuals (N = 12) between water basins were observed in the case of males and also of females not observed to be giving birth. Pihlajavesi acted as a source population for movements, and eight seals moved from there to Haukivesi (N = 2) or to other neighboring water basins (N = 6) while three other seals moved from Haukivesi to neighboring basins. Altogether 11 seals did not return to the basin where they were first observed during the study years. In addition, one more male was re-sighted as having traveled back and forth between Haukivesi and Pihlajavesi in years 2016–2017. The adult females exhibited a high site fidelity for their molting and breeding areas. A total of 19 females observed to give birth (to 29 pups altogether) in the Haukivesi and Pihlajavesi basins during the observation years. These females were re-sighted in the same water basin even during the molt and only one female from the Haukivesi basin was observed in a neighboring water area during one molting season.

We used TEST2.CT to test for trap dependence (trap happiness or trap shyness). No statistically significant trap dependence was observed in any of the Pihlajavesi datasets or in the Haukivesi boat-based survey dataset (p-values from 0.0927 to 0.8111). However, it was notable that all the statistics were negative (from -1.682 to -0.239) pointing together to some degree of trap happiness (positive values would indicate trap shyness).

The median c-hat estimates for all the datasets and accepted models were less than 1.74, and in all cases the 95% confidence intervals included 1. Among the acceptable models the median c-hat for the camera trap datasets was between 0.974 and 0.985 and those for the boat-based survey were between 1.132 and 1.388. In the combined datasets the c-hat values were still acceptable, from 1.546 to 1.740, which indicates that combining data from passive and active observation methods will increase the heterogeneity to some extent. No adjustments were made to the c-hat values, however, to correct possible over-dispersion.

All eight POPAN models fitted nicely only with the Pihlajavesi combined capture history dataset. For the other datasets 3–6 models were acceptable, except that none of the models was acceptable with the Haukivesi 3-year combined dataset (see all the comparison results in S2 Table). Three time points constitute the theoretical minimum for the Jolly-Seber (POPAN) model, but in practice more are needed.

No specific model provided a clear ‘best fit’, but as the highest AICc weighting was between 0.34 and 0.99 (recalculated from AICc weights for acceptable models, Table 3 and S2 Table) [41]. The constant model, *Phi*(·), *p*(·), *pent*(·), *N*, and time-dependent-capture model *Phi*(·), *p*(t), *pent*(·), *N*, were among acceptable models with all datasets (S2 Table).

**Table 3:** Model comparisons (MARK, POPAN model) for the Saimaa ringed seal populations. Individuals photo-identified in the Pihlajavesi (PV, 2013–2017) and Haukivesi (HV, 2010–2017) basins. Separate results are presented for the various models and for the combination of two observation methods (Pihlajavesi) or only the boat-based survey (Haukivesi).

			Delta	AICc	Mark rpt		
	Model	AICc	AICc	Weights	No. Par	Deviance	
PV	2: Phi(·)p(·)pent(t)N(·)	402.602	0	0.498	7	-277.1	X
Combined	1: Phi(·)p(·)pent(·)N(·)	404.478	1.876	0.195	4	-268.96	X
N obs 115	4: Phi(·)p(t)pent(t)N(·)	405.243	2.641	0.133	10	-280.87	X
2013–17	3: Phi(·)p(t)pent(·)N(·)	406.604	4.002	0.067	8	-275.22	X
	6: Phi(t)p(·)pent(t)N(·)	407.228	4.626	0.049	10	-278.89	X
	5: Phi(t)p(·)pent(·)N(·)	408.411	5.809	0.027	7	-271.29	X
	8: Phi(t)p(t)pent(t)N(·)	408.868	6.266	0.022	12	-281.61	X
	7: Phi(t)p(t)pent(·)N(·)	410.715	8.112	0.009	10	-275.4	X
HV	3: Phi(·)p(t)pent(·)N(·)	413.866	0	0.869	11	-111.99	X
Boat	4: Phi(·)p(t)pent(t)N(·)	418.120	4.254	0.104	14	-114.58	
survey	7: Phi(t)p(t)pent(·)N(·)	421.892	8.027	0.016	16	-115.5	
N obs 68	1: Phi(·)p(·)pent(·)N(·)	423.079	9.214	0.009	4	-87.603	X
2010–17	8: Phi(t)p(t)pent(t)N(·)	425.920	12.05	0.002	19	-118.69	
	5: Phi(t)p(·)pent(·)N(·)	427.215	13.35	0.001	8	-92.004	
	2: Phi(·)p(·)pent(t)N(·)	432.185	18.32	0.000	10	-91.436	X
	6: Phi(t)p(·)pent(t)N(·)	437.099	23.23	0.000	14	-95.605	

X Model was acceptable.

In both water basins the estimated survival rates for the seals were high (0.813–0.929) and the re-capture probabilities were also high (0.362–0.750) (Table 4 and S3 Table). All model comparison results for the various datasets are presented in S3 Table. The re-capture probabilities (boat-based survey data in Haukivesi basin) correlated strongly with the observation effort measured as the boat-based survey days (Pearson r = 0.86, p = 0.0288, n = 6, years 2011–2016) (Table 4 and S3 Table).

**Table 4:** Model-averaged real parameter estimates of acceptable Jolly-Seber (POPAN) models: Apparent survival (Phi), re-capture probability (p) and entrance probability (pent) are presented. Separate results are quoted for A) combined data from the two data collection methods in Pihlajavesi (2013–2017) and B) the boat-based survey data for Haukivesi (2010–2017). Parameters marked with grey are reliably estimated. Standard errors (SE, delta method) and 95% confidence intervals are presented (derived from Beta parameter estimates with inverse logit link).

A) Pihlajavesi, 2013–17							B) Haukivesi, 2010–17			
Combined	N obs 115						Boat survey, N obs 68			
			95% C. I.						95% C. I.	
Parameter	Estimate*	SE	Lower	Upper			Estimate¤	SE	Lower	Upper
Phi1	0.888	0.0334	0.805	0.939		Phi1	0.929	0.0220	0.872	0.961
Phi2	0.887	0.0326	0.806	0.937		Phi2	0.929	0.0220	0.872	0.961
Phi3	0.891	0.0357	0.799	0.944		Phi3	0.929	0.0220	0.872	0.961
Phi4	0.874	0.0635	0.691	0.955		Phi4	0.929	0.0220	0.872	0.961
p1	0.764	0.1116	0.491	0.916		Phi5	0.929	0.0220	0.872	0.961
p2	0.710	0.0571	0.587	0.808		Phi6	0.929	0.0220	0.872	0.961
p3	0.724	0.0486	0.619	0.808		Phi7	0.929	0.0220	0.872	0.961
p4	0.743	0.0590	0.612	0.842		p1	0.508	0.1313	0.269	0.743
p5	0.716	0.0681	0.567	0.830		p2	0.378	0.0947	0.216	0.572
pent1	0.172	0.0744	0.069	0.366		p3	0.631	0.0929	0.438	0.789
pent2	0.084	0.0521	0.024	0.257		p4	0.469	0.0808	0.318	0.625
pent3	0.218	0.0704	0.110	0.385		p5	0.362	0.0757	0.230	0.519
pent4	0.088	0.0488	0.029	0.241		p6	0.744	0.0728	0.578	0.860
						p7	0.750	0.0789	0.568	0.873
						p8	0.647	0.0851	0.469	0.792
						pent1	0.079	0.0147	0.054	0.113
						pent2	0.079	0.0147	0.054	0.113
						pent3	0.079	0.0147	0.054	0.113
						pent4	0.079	0.0147	0.054	0.113
						pent5	0.079	0.0147	0.054	0.113
						pent6	0.079	0.0147	0.054	0.113
						pent7	0.079	0.0147	0.054	0.113

* All eight models included

¤ Three models included: due to Akaike's weight only the. Phi.dot.p.time.pent.dot model has any practical effect on the parameters.

The population growth during the observation period may be anticipated both in the number of animals observed and in the population size estimates. The Pihlajavesi population estimate increased by 22 individuals in three years when the model was providing reliable estimates and Haukivesi had a population rise of 14 individuals during six years (Table 5). This corresponds to growth rates of about 15% and 3%, although the first of these numbers might not be biologically possible even though the numbers themselves would be correct. It should be noted that no increase in the estimated population size was observed when the passive camera trap was used (Table 5).

**Table 5:** Model-averaged population size estimates of the acceptable Jolly-Seber (POPAN) models. Separate results are presented for the camera trap, boat-based survey and combined data for the two methods in the Pihlajavesi (2013–2017) and Haukivesi (2010–2012) basins using the camera trap method and for a longer period using boat-based survey (2010–2017). Years marked with grey are reliably estimated. Standard errors (SE, delta method) and 95% confidence intervals are presented.

							95% C. I. Lower Upper	
Lake	Observation method	Year	N obs.	Parameter	Estimate	SE		
Pihlajavesi		2013	33	N-hat	73.86	15.200	49.55	110.09
	Camera trap	2014	36		72.50	10.364	54.86	95.80
		2015	30		71.06	7.697	57.51	87.82
		2016	51		71.26	8.019	57.20	88.78
		2017	33		72.01	10.914	53.59	96.75
		2013–17	92	N*-hat	116.00	7.318	102.52	131.26
Pihlajavesi		2013	32	N-hat	55.77	9.939	39.44	78.87
	Boat survey	2014	32		67.10	7.899	53.32	84.45
		2015	42		78.65	7.421	65.40	94.59
		2016	57		91.22	9.348	74.66	111.45
		2017	52		98.29	10.662	79.52	121.50
		2013–17	107	N*-hat	131.22	6.933	118.32	145.53
Pihlajavesi		2013	41	N-hat	54.37	9.515	38.68	76.42
	Combined	2014	48		69.53	6.981	57.14	84.61
		2015	52		72.10	6.717	60.09	86.51
		2016	73		91.17	7.755	77.19	107.67
		2017	62		90.59	9.072	74.48	110.19
		2013–17	115	N*-hat	128.16	4.310	119.99	136.89
Haukivesi		2010	23	N-hat	28.62	8.712	15.97	51.29
	Camera trap	2011	21		33.57	6.438	23.13	48.72
		2012	31		35.81	7.660	23.65	54.21
		2010–12	44	N*-hat	49.61	3.631	42.99	57.25
Haukivesi		2010	17	N-hat	33.47	7.848	21.27	52.67
	Boat survey	2011	14		36.91	6.274	26.52	51.38
		2012	26		40.11	4.953	31.52	51.05
		2013	21		43.08	4.012	35.91	51.69
		2014	16		45.84	3.625	39.26	53.51
		2015	36		48.40	3.862	41.40	56.58
		2016	37		50.78	4.550	42.61	60.50
		2017	34		52.98	5.470	43.30	64.83
		2010–17	68	N*-hat	75.85	3.336	69.58	82.67
Haukivesi								
	Combined	No result						

## Discussion

Socioeconomic pressure from local people and the negative effects of climate change call for new or alternative tools for increasing transparency and reliability in the monitoring of Saimaa ringed seal populations. We propose here the first systematic and reproducible photo-ID-based mark-recapture approach for estimating the size of the population of this endangered seal. This non-invasive method provides an urgently needed scientifically based census approach which is less dependent on expert knowledge and therefore more acceptable to various interest groups. In addition, given that the current snow lair census method is heavily depending on the snow and ice situation, which is less predictable under rapidly changing winter conditions, our approach is also suitable for use under changing climatic conditions. Based on a long-term scenario, it is estimated that the climate in Finland will continue to warm up by as much as 2–7°C by the 2080s, which will greatly affect weather conditions, reducing the amount of snow and shortening the winter season [51–53]. Mark-recapture censuses can be carried out without being dependent on snow conditions or the amount of lake ice, and they are relatively cost-effective and more inclusive in the future, since the photo-ID approach will allow the public to take part in collecting the image data. A Saimaa ringed seal image gallery (www.norppagalleria.fi) has recently been published, and tourists and other outdoor people are being encouraged to submit images acquired on seal watching tours for the benefit of photo-ID studies. Thus, through methodical data collection and processing, this approach could greatly increase the transparency and reliability of ringed seal conservation and monitoring.

The camera trap approach is a non-invasive method for obtaining reliable mark-recapture data and it has been widely used in studies of rare or elusive terrestrial carnivores in order to minimize the disturbance caused by monitoring efforts (e.g., [54–56]). Although mark-recapture population estimation methods have been used recently with a few pinniped species [31–35, 57], and have been in use for a longer time with cetaceans (e.g.,[58–60]), this is to the best of our knowledge the first project using camera-trapping for marine mammals to take place on such a large scale and also the first mark-recapture project to provide population estimates for a species of ringed seal.

### Model assumption validation

The photo-ID method is systematic and reliable, but statistical mark-recapture models entail assumptions that need to be taken into account when interpreting the results. When considering the assumption behind the POPAN model, photo-ID, as a “mark-recapture method”, enables sampling to be instantaneous, after which “the animals are released immediately”. The observation season is relatively short (5–9 weeks annually) compared with a one-year occasion interval and observation can therefore be regarded as instantaneous. Moreover, there is no possibility that photo-ID could affect the behavior or survival rate of the seals, since our sampling method is non-invasive. As the pups are not included in the data, the older individuals should have the same survival probability, because the survival rate is known to stabilize after the first critical year of life [23, 61], or more precisely, after the first 15 months, as recent results have pointed out [62]. The high survival rate of adult seals was also seen in the survival of over one-year-old seals (around 81–93%) in our study. Also, our re-sighting percentages (over 50% in the total data) and re-capture probabilities (0.362–0.750) were both of the same magnitude as those reported in other mark-recapture studies performed on pinnipeds [31, 33–34,57,63]. These high values reflect the good quality of our population estimates [38]. It has been suggested that capture probabilities greater than 0.3 are reliable and indicate the accuracy of mark-recapture estimates [64, 65]. High re-capture probability observed in this study can be explained by seals’ high site fidelity for molting areas [36] and/or by the fact that these intensive surveys were carried out on small seal populations with a limited habitat. The pelage patterns of the seals are life-long, permanent and easily visible properties, which reduces the possibility of misidentifying the individuals.

The data collection methods used here differ in their re-capture probabilities, as camera trapping is a passive method that captures the same local individuals many times, while the boat-based survey method as an active method may capture different individuals better also on the edge areas. For these reasons, individual seals in our study areas have unequal capture probabilities to some an unknown extent, and this will introduce individual heterogeneity into the data, which is known to affect both population parameter estimates and their uncertainty estimates [66]. A simulation study with two bird species [66] has shown that various kinds of heterogeneity pattern (trap happiness, trap shyness, sex, age class) can result in underestimation of both survival and the corresponding uncertainty estimates. In this study, the trap dependence was tested and some effect towards trap happiness was observed, as can be expected with species showing high site fidelity, so that the same individuals are often re-sighted in the same places [67]. Saimaa ringed seals are known to exhibit high site fidelity for their breeding [17, 68] and molting areas [12, 36], which was also confirmed in the present study. Breeding site fidelity is especially notable in the females [68].

As a statistically significant transient effect was observed, this should be taken into account in future population models when surveying the whole Lake Saimaa basin since it has been pointed out that sub-adult seals may move to the edges of the breeding areas, which would support this transient effect [69]. This effect could be introduced into the Cormack–Jolly–Seber models and the estimation of survival in future studies, but it is not applicable to the POPAN model as used here (see [30]). In addition, the survey effort can be incorporated into the models as a covariate parameter. One interesting further modeling perspective would be to analyze the female and male data separately to determine the effect of sex on the survival and population size results.

### Monitoring implications

Our recommendation is to use of both camera traps and boat-based surveys when collecting image data for mark-recapture analyses. Combining these datasets provides more identified individuals and re-sightings than either dataset alone, and will increase re-capture probability and thereby improve the accuracy of the population estimates. Both data collection methods entail both benefits and weaknesses. Camera traps provide a large amount of data to handle and are more labor-intensive than boat-based surveys, since the memory card needs to be changed regularly. Camera trapping also requires more knowledge of both seals and game cameras, whereas boat-based surveys can also be carried out by casual members of the public. Our results obtained for Haukivesi show that even a low annual boat-based survey effort with long enough study period (sufficient amount of time points) can be useful for photo-ID purposes and can provide a mark-recapture estimate. Camera trapping may favor individuals with high site fidelity, whereas boat-based surveys will also allow us to capture those seals that do not return to the same haul-out site after the first occurrence. Camera traps provide high quality close-up images that are ideal for identification purposes and they also record other valuable data such as sex, age class, social interaction and behavioral information [36], whereas boat-based surveys provide less information and produces images of variable quality. We observed that camera traps also allow us to capture shy individuals and seals that haul out in sheltered places that we have not been able to capture during boat-based surveys. There is some individual variation in seal behavior, in that some will leave their haul-out site when the approaching boat is still far away [70]. The fact that the annual population size estimates did not increase when only the passive camera trap data were used may be due to the seals’ site fidelity if the haul-out sites are more or less fully occupied, i.e. the maximum number of animals is reached. Boat-based surveys represent an active observation method, and thus pointed to increasing population size, as was also seen when the camera trap and boat-based observations were combined.

Although our mark-recapture population estimates are quite the same magnitude as the official seal numbers for the Pihlajavesi and Haukivesi basins produced by the currently used lair census method [18], they are systematically lower. Time may have an effect on the results, since our results showed that in practice data for at least four successive molting seasons are needed for reliable mark-recapture estimates. The population sizes for 2016 estimated using the lair census method were 104–128 individuals in the Pihlajavesi basin and 65–86 in Haukivesi [18]. When comparing these with our estimates for the same year, when we had the best areal coverage of both basins, the photo-ID based results are still respectively 16–25% and 29–34% lower than the numbers given by the lair census method. This can still be explained by the smaller spatial coverage of the photo-ID study as compared with the lair censuses. Similarly variations in effort and survey intensity may have affected the results, especially in the Haukivesi basin. Our estimates indicated population growth during the years concerned, which is in accordance with the lair census results. However, rate of this growth (3–15%) estimated in ours study was higher than in the estimates obtained by the lair census method (around 3%) [14], which can also be explained by increased efforts (both temporally and spatially) in the course of the photo-ID years. Moreover, the results showed a transient effect for the Pihlajavesi basin, which was also confirmed by observations of the movements of individuals. This highlights the role of Pihlajavesi basin, which has the best pup production in the whole of Lake Saimaa, as a source of population for other regions. On the other hand, the transient effect could also indicate that large proportions of the individuals were dismissed over the years and that some seals were encountered only once. This could be partly a result of hidden mortality among juveniles, which also continues into early sub-adulthood [62].

It is significant that the current lair census method [18] requires an expert opinion, and the result will most probably be different if another expert is consulted, which means that these estimates may include a significant degree of uncertainty in the long term. In addition, estimates based on snow lair censuses may become less reliable as the snow cover diminishes. Population estimates based on lair and pup numbers may suffer from some serious biases due to climatic factors [6], hidden mortality of individuals [62] and the behavior patterns of ringed seals [70]. In the long run, with increased datasets, the mark-recapture approach will be repeatable and transparent, so that it can be used as a basis for objective conservation decisions. Moreover, it will allow estimation of other important population parameters such as survival, population growth rate and reproductive capacity in addition to the size of the population (e.g., [45, 71–72]).

### Future perspectives

Climate change constitutes a major challenge for the monitoring and conservation of the Saimaa ringed seal (e.g., [5–7]). Our current findings, along with those reported earlier by Koivuniemi et al. [36], confirm that photo-ID mark-recapture techniques represent an effective and valuable approach for monitoring this endangered subspecies for conservation purposes under changing climatic conditions. This approach enables us to follow individual seals over their lifespan and expand our observations on both the individual and the population level. While the study presented here focused on the main breeding areas of the Saimaa ringed seal, future research plans intend to expand this effort and perform mark-recapture analyses over the whole of Lake Saimaa. We propose to use the snow lair census technique in parallel with the mark-recapture approach and maximize the use of both types of data (e.g. through a Bayesian approach). Especially the combination of a pup lair census and a photo-ID-based mark-recapture analysis would facilitate more accurate estimates of annual juvenile survival rates by comparing observed births with model-based birth rates (estimates of juvenile survival are currently based mainly on observed mortality). With photo-ID data collected systematically across a broader geographic region and an even longer time period (several years), the resulting multi-year dataset, together with the photo-ID mark-recapture approach, should further improve the accuracy of the estimated population parameters. It also improves the precision of the distribution pattern presented for the Saimaa ringed seal population, and is likely to facilitate more in-depth analyses of the social structure of the population and its long-term demographic trajectory, all of which are crucial for maximizing the effectiveness of current and future conservation efforts.

## Supporting information

S1 Table Re-capture histories of Saimaa ringed seals and observed numbers and percentages of seals identified. Individuals re-sighted using the camera traps, boat-based survey and their combination in A) Pihlajavesi basin (2013–2017), B) Haukivesi basin (2010–2012) and C) based only on the boat-based survey in Haukivesi basin (2010–2017).
(DOCX)

S2 Table Model comparison (MARK, POPAN model) for the population of the Saimaa ringed seal. Individuals over one-year-old photo-identified in the Pihlajavesi basin (PV, 2013–2017) and the Haukivesi basin (HV, 2010–2012 camera trap and 2010–2017 boat-based survey). Separate results are presented for the various models and for the two observation methods and their combination.
(DOCX)

S3 Table Model-averaged real parameter estimates of acceptable Jolly-Seber (POPAN) models: apparent survival (Phi), re-capture probability (p) and entrance probability (pent) are presented. Separate results are presented for 1A) the camera traps, 2A) the boat-based surveys and 3A) their combined data in Pihlajavesi (2013–2017) and for the 1B) camera traps (2010–2013) and 2B) boat-based surveys (2010–2017) in Haukivesi. Parameters marked with grey are reliably estimated. Standard errors (SE, delta method) and 95% confidence intervals are presented (derived from Beta parameter estimates with inverse logit link).
(DOCX)
