# Supplementary material for: A mark–recapture approach for estimating population size of the endangered ringed seal (Phoca hispida saimensis)
Source: PLoS One. 2019 Mar 22;14(3):e0214269. doi: 10.1371/journal.pone.0214269 (PMC6430510; doi:10.1371/journal.pone.0214269)
Supplement: S1 Table — Individuals re-sighted using the camera traps, boat-based survey and their combination in A) Pihlajavesi basin (2013–2017), B) Haukivesi basin (2010–2012) and C) based only on the boat-based survey in Haukivesi basin (2010–2017). (DOCX) [file pone.0214269.s001.docx]

**S1 Table. Re-capture histories of Saimaa ringed seals and observed numbers and percentages of seals identified*.*** Individuals re-sighted using the camera traps, boat-based survey and their combination in A) Pihlajavesi basin (2013-2017), B) Haukivesi basin (2010-2012) and C) based only on the boat-based survey in Haukivesi basin (2010-2017).

|  | 1. Pihlajavesi | |  |  |  | Observed numbers and percentages  seals identified | | |
| --- | --- | --- | --- | --- | --- | --- | --- | --- |
| Capture history* | Camera trap | Boat survey | Combined |  |  |  |  |  |
|  |  |  |  |  |  | Camera trap | Boat survey | Combined |
| _11111 | 4 | 6 | 14 |  | 1. year | 33 | 32 | 41 |
| _11110 | 2 | 1 | 3 |  | 2. year | 36 | 32 | 48 |
| _11101 |  | 1 | 1 |  | 3. year | 30 | 42 | 52 |
| _11100 | 1 |  |  |  | 4. year | 51 | 57 | 73 |
| _11011 |  | 1 | 2 |  | 5. year | 33 | 52 | 62 |
| _11010 | 3 | 1 | 2 |  | all | 92 | 107 | 115 |
| _11001 | 1 | 3 | 2 |  |  |  |  |  |
| _11000 | 2 |  |  |  | once | 42 | 51 | 42 |
| _10111 | 1 | 3 | 3 |  | twice | 23 | 25 | 28 |
| _10110 | 3 | 2 | 4 |  | three times | 17 | 16 | 16 |
| _10101 | 1 |  | 1 |  | four times | 6 | 9 | 15 |
| _10100 | 1 | 3 | 2 |  | five times | 4 | 6 | 14 |
| _10011 | 1 | 1 |  |  |  |  |  |  |
| _10010 | 1 | 1 |  |  | at least twice | 50 | 56 | 73 |
| _10001 | 2 | 2 | 1 |  | at least 3 t | 27 | 31 | 45 |
| _10000 | 10 | 7 | 6 |  | at least 4 t | 10 | 15 | 29 |
| _01111 | 3 | 3 | 6 |  |  |  |  |  |
| _01110 | 3 | 3 | 2 |  | once % | 45.7 | 47.7 | 36.5 |
| _01101 | 1 |  |  |  | twice % | 25.0 | 23.4 | 24.3 |
| _01100 |  | 2 | 4 |  | three times% | 18.5 | 15.0 | 13.9 |
| _01011 | 1 | 2 | 1 |  | four times% | 6.5 | 8.4 | 13.0 |
| _01010 | 5 | 1 | 3 |  | five times % | 4.3 | 5.6 | 12.2 |
| _01001 | 1 | 1 |  |  |  |  |  |  |
| _01000 | 9 | 7 | 8 |  | at least twice % | 54.3 | 52.3 | 63.5 |
| _00111 | 2 | 4 | 4 |  | at least 3 t % | 29.3 | 29.0 | 39.1 |
| _00110 | 2 | 4 | 3 |  | at least 4 t % | 10.9 | 14.0 | 25.2 |
| _00101 | 1 | 4 | 2 |  |  |  |  |  |
| _00100 | 5 | 6 | 3 |  |  |  |  |  |
| _00011 | 8 | 7 | 13 |  |  |  |  |  |
| _00010 | 12 | 17 | 13 |  |  |  |  |  |
| _00001 | 6 | 14 | 12 |  |  |  |  |  |
|  |  |  |  |  |  |  |  |  |

*Capture history: in which years an individual was observed during the five-year study period. The maximum number of capture history combinations is 31 i.e. 2^5^-1.

|  | Observed numbers and percentages  seals identified | | |
| --- | --- | --- | --- |
|  |  |  |  |
|  | Camera trap | Boat survey | Combined |
| 1. year | 23 | 17 | 29 |
| 2. year | 21 | 14 | 24 |
| 3. year | 31 | 26 | 40 |
| all | 44 | 35 | 51 |
|  |  |  |  |
| once | 21 | 18 | 19 |
| twice | 15 | 12 | 22 |
| three times | 8 | 5 | 10 |
|  |  |  |  |
| at least twice | 23 | 17 | 32 |
|  |  |  |  |
| once % | 47.7 | 51.4 | 37.3 |
| twice % | 34.1 | 34.3 | 43.1 |
| three times% | 18.2 | 14.3 | 19.6 |
| at least twice % | 52.3 | 48.6 | 62.7 |

|  | B) Haukivesi | |  |
| --- | --- | --- | --- |
| Capture history* | Camera trap | Boat survey** | Combined |
|  |  |  |  |
| _111 | 8 | 5 | 10 |
| _110 | 3 |  | 2 |
| _101 | 5 | 8 | 11 |
| _100 | 7 | 4 | 6 |
| _011 | 7 | 4 | 9 |
| _010 | 3 | 5 | 3 |
| _001 | 11 | 9 | 10 |

*Capture history: in which years an individual was observed during the three-year study period. The maximum number of capture history combinations is 8 i.e. 2^3^-1.

** Three year boat survey data is presented here for consistence, POPAN-model is fitted for eight year data.

|  | 1. Haukivesi |  | Observed numbers and percentages  seals identified | |
| --- | --- | --- | --- | --- |
| Capture history* | Boat survey |  |  |  |
|  |  |  |  | Boat survey |
| _11111111 | 2 |  | 1. year | 17 |
| _11111100 | 1 |  | 2. year | 14 |
| _11110101 | 1 |  | 3. year | 26 |
| _11100000 | 1 |  | 4. year | 21 |
| _10111111 | 1 |  | 5. year | 16 |
| _10110111 | 2 |  | 6. year | 36 |
| _10110000 | 1 |  | 7. year | 37 |
| _10101000 | 1 |  | 8.year | 34 |
| _10100100 | 1 |  | all | 68 |
| _10100000 | 2 |  |  |  |
| _10001101 | 1 |  | once | 18 |
| _10000110 | 1 |  | twice | 11 |
| _10000000 | 2 |  | three times | 18 |
| _01110101 | 1 |  | four times | 8 |
| _01110010 | 1 |  | five times | 5 |
| _01101100 | 1 |  | six times | 5 |
| _01100111 | 1 |  | seven times | 1 |
| _01010111 | 1 |  | eight times | 2 |
| _01001110 | 1 |  |  |  |
| _01000111 | 1 |  | at least twice | 50 |
| _01000011 | 1 |  | at least 3 t | 39 |
| _01000010 | 1 |  | at least 4 t | 21 |
| _00111111 | 1 |  | at least 5 t | 13 |
| _00111110 | 1 |  | at least 6 t | 8 |
| _00101111 | 1 |  | at least 7 t | 3 |
| _00100110 | 1 |  |  |  |
| _00100100 | 1 |  | once % | 26.5 |
| _00100011 | 1 |  | twice % | 16.2 |
| _00100001 | 1 |  | three times% | 26.5 |
| _00100000 | 2 |  | four times% | 11.8 |
| _00011101 | 1 |  | five times % | 7.4 |
| _00011100 | 1 |  | six times % | 7.4 |
| _00010111 | 1 |  | seven times % | 1.5 |
| _00010110 | 1 |  | eight times % | 2.9 |
| _00010011 | 1 |  |  |  |
| _00010010 | 1 |  | at least twice % | 73.5 |
| _00010000 | 2 |  | at least 3 t % | 57.4 |
| _00001111 | 1 |  | at least 4 t % | 30.9 |
| _00001110 | 1 |  | at least 5 t % | 19.1 |
| _00001011 | 1 |  | at least 6 t % | 11.8 |
| _00001000 | 1 |  | at least 7 t % | 4.4 |
| _00000111 | 5 |  |  |  |
| _00000110 | 2 |  |  |  |
| _00000100 | 3 |  |  |  |
| _00000011 | 3 |  |  |  |
| _00000010 | 3 |  |  |  |
| _00000001 | 5 |  |  |  |

*Capture history: in which years an individual was observed during the eight-year study period. The maximum number of capture history combinations is 255 i.e. 2^8^-1.
