# Supplementary material for: A mark–recapture approach for estimating population size of the endangered ringed seal (Phoca hispida saimensis)
Source: PLoS One. 2019 Mar 22;14(3):e0214269. doi: 10.1371/journal.pone.0214269 (PMC6430510; doi:10.1371/journal.pone.0214269)
Supplement: S2 Table — Individuals over one-year-old photo-identified in the Pihlajavesi basin (PV, 2013–2017) and the Haukivesi basin (HV, 2010–2012 camera trap and 2010–2017 boat-based survey). Separate results are presented for the various models and for the two observation methods and their combination. (DOCX) [file pone.0214269.s002.docx]

**S2 Table. Model comparison (MARK, POPAN model) for the population of the Saimaa ringed seal*.*** Individuals over one-year-old photo-identified in the Pihlajavesi basin (PV, 2013-2017) and the Haukivesi basin (HV, 2010-2012 camera trap and 2010-2017 boat-based survey). Separate results are presented for the various models and for the two observation methods and their combination.

|  |  |  | Delta | AICc | Mark rpt |  |  |
| --- | --- | --- | --- | --- | --- | --- | --- |
| Observation method | Model | AICc | AICc | Weights | Num. Par | Deviance |  |
| PV | 6: Phi(t)p(·)pent(t)N(·) | 341.273 | 0 | 0.303 | 8 | -224.04 |  |
| Camera | 2: Phi(·)p(·)pent(t)N(·) | 341.852 | 0.58 | 0.227 | 6 | -219.11 |  |
| trap | 4: Phi(·)p(t)pent(t)N(·) | 342.467 | 1.19 | 0.167 | 9 | -225.06 |  |
| N obs 92 | 3: Phi(·)p(t)pent(·)N(·) | 342.538 | 1.27 | 0.161 | 8 | -222.78 | X |
| 2013-17 | 5: Phi(t)p(·)pent(·)N(·) | 344.615 | 3.34 | 0.057 | 6 | -216.35 |  |
|  | 8: Phi(t)p(t)pent(t)N(·) | 345.685 | 4.41 | 0.033 | 11 | -226.35 |  |
|  | 7: Phi(t)p(t)pent(·)N(·) | 345.995 | 4.72 | 0.029 | 10 | -223.77 | X |
|  | 1: Phi(·)p(·)pent(·)N(·) | 346.401 | 5.13 | 0.023 | 4 | -210.31 | X |
| PV | 1: Phi(·)p(·)pent(·)N(·) | 362.361 | 0 | 0.683 | 4 | -273.54 | X |
| Boat | 2: Phi(·)p(·)pent(t)N(·) | 365.313 | 2.95 | 0.156 | 7 | -276.94 | X |
| survey | 3: Phi(·)p(t)pent(·)N(·) | 366.952 | 4.59 | 0.069 | 8 | -277.46 | X |
| N obs 107 | 5: Phi(t)p(·)pent(·)N(·) | 367.249 | 4.89 | 0.059 | 7 | -275 |  |
| 2013-17 | 4: Phi(·)p(t)pent(t)N(·) | 370.118 | 7.76 | 0.014 | 10 | -278.67 |  |
|  | 6: Phi(t)p(·)pent(t)N(·) | 371.110 | 8.75 | 0.009 | 10 | -277.68 | X |
|  | 7: Phi(t)p(t)pent(·)N(·) | 371.148 | 8.79 | 0.008 | 10 | -277.64 | X |
|  | 8: Phi(t)p(t)pent(t)N(·) | 374.079 | 11.7 | 0.002 | 12 | -279.18 | X |
| PV | 2: Phi(·)p(·)pent(t)N(·) | 402.602 | 0 | 0.498 | 7 | -277.1 | X |
| Combined | 1: Phi(·)p(·)pent(·)N(·) | 404.478 | 1.88 | 0.195 | 4 | -268.96 | X |
| N obs 115 | 4: Phi(·)p(t)pent(t)N(·) | 405.243 | 2.64 | 0.133 | 10 | -280.87 | X |
| 2013-17 | 3: Phi(·)p(t)pent(·)N(·) | 406.604 | 4 | 0.067 | 8 | -275.22 | X |
|  | 6: Phi(t)p(·)pent(t)N(·) | 407.228 | 4.63 | 0.049 | 10 | -278.89 | X |
|  | 5: Phi(t)p(·)pent(·)N(·) | 408.411 | 5.81 | 0.027 | 7 | -271.29 | X |
|  | 8: Phi(t)p(t)pent(t)N(·) | 408.868 | 6.27 | 0.022 | 12 | -281.61 | X |
|  | 7: Phi(t)p(t)pent(·)N(·) | 410.715 | 8.11 | 0.009 | 10 | -275.4 | X |
| HV | 5: Phi(t)p(·)pent(·)N(·) | 94.107 | 0 | 0.284 | 4 | -77.685 |  |
| Camera | 4: Phi(·)p(t)pent(t)N(·) | 94.122 | 0.015 | 0.282 | 5 | -79.969 |  |
| trap | 6: Phi(t)p(·)pent(t)N(·) | 96.098 | 1.991 | 0.105 | 5 | -77.992 |  |
| N obs 44 | 1: Phi(·)p(·)pent(·)N(·) | 96.364 | 2.257 | 0.092 | 4 | -75.428 | X |
| 2010-12 | 8: Phi(t)p(t)pent(t)N(·) | 96.464 | 2.357 | 0.088 | 6 | -79.992 | X |
|  | 7: Phi(t)p(t)pent(·)N(·) | 97.340 | 3.233 | 0.056 | 6 | -79.116 |  |
|  | 3: Phi(·)p(t)pent(·)N(·) | 97.455 | 3.348 | 0.053 | 6 | -79.001 | X |
|  | 2: Phi(·)p(·)pent(t)N(·) | 98.095 | 3.988 | 0.039 | 5 | -75.995 | X |
| HV | 3: Phi(·)p(t)pent(·)N(·) | 413.866 | 0 | 0.869 | 11 | -111.99 | X |
| Boat | 4: Phi(·)p(t)pent(t)N(·) | 418.120 | 4.254 | 0.104 | 14 | -114.58 |  |
| survey | 7: Phi(t)p(t)pent(·)N(·) | 421.892 | 8.027 | 0.016 | 16 | -115.5 |  |
| N obs 68 | 1: Phi(·)p(·)pent(·)N(·) | 423.079 | 9.214 | 0.009 | 4 | -87.603 | X |
| 2010-17 | 8: Phi(t)p(t)pent(t)N(·) | 425.920 | 12.05 | 0.002 | 19 | -118.69 |  |
|  | 5: Phi(t)p(·)pent(·)N(·) | 427.215 | 13.35 | 0.001 | 8 | -92.004 |  |
|  | 2: Phi(·)p(·)pent(t)N(·) | 432.185 | 18.32 | 0.000 | 10 | -91.436 | X |
|  | 6: Phi(t)p(·)pent(t)N(·) | 437.099 | 23.23 | 0.000 | 14 | -95.605 |  |
| HV | 4: Phi(·)p(t)pent(t)N(·) | 107.300 | 0 | 0.282 | 5 | -89.164 |  |
| Combined | 8: Phi(t)p(t)pent(t)N(·) | 107.300 | 0.000 | 0.282 | 5 | -89.164 |  |
| N obs 51 | 3: Phi(·)p(t)pent(·)N(·) | 108.039 | 0.739 | 0.195 | 5 | -88.424 |  |
| 2010-12* | 7: Phi(t)p(t)pent(·)N(·) | 108.039 | 0.739 | 0.195 | 5 | -88.424 |  |
|  | 1: Phi(·)p(·)pent(·)N(·) | 112.449 | 5.150 | 0.021 | 3 | -79.594 |  |
|  | 5: Phi(t)p(·)pent(·)N(·) | 113.480 | 6.180 | 0.013 | 4 | -80.749 |  |
|  | 2: Phi(·)p(·)pent(t)N(·) | 114.184 | 6.884 | 0.009 | 4 | -80.045 |  |
|  | 6: Phi(t)p(·)pent(t)N(·) | 115.557 | 8.257 | 0.005 | 5 | -80.907 |  |

X Model was functioning

* None of the models (1-8) were acceptable.
