# Supplementary material for: A mark–recapture approach for estimating population size of the endangered ringed seal (Phoca hispida saimensis)
Source: PLoS One. 2019 Mar 22;14(3):e0214269. doi: 10.1371/journal.pone.0214269 (PMC6430510; doi:10.1371/journal.pone.0214269)
Supplement: S3 Table — Separate results are presented for 1A) the camera traps, 2A) the boat-based surveys and 3A) their combined data in Pihlajavesi (2013–2017) and for the 1B) camera traps (2010–2013) and 2B) boat-based surveys (2010–2017) in Haukivesi. Parameters marked with grey are reliably estimated. Standard errors (SE, delta method) and 95% confidence intervals are presented (derived from Beta parameter estimates with inverse logit link). (DOCX) [file pone.0214269.s003.docx]

**S3 Table. Model-averaged real parameter estimates of acceptable Jolly-Seber (POPAN) models:** apparent survival (Phi), re-capture probability (p) and entrance probability (pent) are presented. Separate results are presented for 1A) the camera traps, 2A) the boat-based surveys and 3A) their combined data in Pihlajavesi (2013-2017) and for the 1B) camera traps (2010-2013) and 2B) boat-based surveys (2010-2017) in Haukivesi. Parameters marked with grey are reliably estimated. Standard errors (SE, delta method) and 95% confidence intervals are presented (derived from Beta parameter estimates with inverse logit link).

| 1A) Pihlajavesi, years 2013-17 | | | |  |  | 2A) Pihlajavesi, years 2013-17 | | | | |
| --- | --- | --- | --- | --- | --- | --- | --- | --- | --- | --- |
| Camera trap, N obs 92 | | |  |  |  | Boat survey, N obs 107 | | | |  |
|  |  |  | 95% C. I. | |  |  |  | 95% C. I. | | |
| Parameter | Estimate* | SE | Lower | Upper |  | Estimate* | SE | Lower | | Upper |
| Phi1 | 0.900 | 0.0397 | 0.791 | 0.955 |  | 0.849 | 0.0563 | 0.704 | | 0.930 |
| Phi2 | 0.901 | 0.0394 | 0.793 | 0.955 |  | 0.845 | 0.0568 | 0.700 | | 0.928 |
| Phi3 | 0.901 | 0.0407 | 0.788 | 0.957 |  | 0.866 | 0.0694 | 0.667 | | 0.954 |
| Phi4 | 0.900 | 0.0452 | 0.771 | 0.960 |  | 0.872 | 0.0638 | 0.690 | | 0.955 |
| p1 | 0.553 | 0.0690 | 0.417 | 0.681 |  | 0.450 | 0.1021 | 0.267 | | 0.647 |
| p2 | 0.543 | 0.0584 | 0.428 | 0.653 |  | 0.498 | 0.0855 | 0.336 | | 0.659 |
| p3 | 0.549 | 0.0524 | 0.445 | 0.648 |  | 0.437 | 0.0764 | 0.297 | | 0.588 |
| p4 | 0.557 | 0.0571 | 0.444 | 0.664 |  | 0.687 | 0.0999 | 0.469 | | 0.845 |
| p5 | 0.549 | 0.0570 | 0.437 | 0.656 |  | 0.468 | 0.0854 | 0.310 | | 0.632 |
| pent1 | 0.133 | 0.0426 | 0.069 | 0.240 |  | 0.087 | 0.0312 | 0.042 | | 0.170 |
| pent2 | 0.143 | 0.0330 | 0.090 | 0.220 |  | 0.087 | 0.0312 | 0.042 | | 0.170 |
| pent3 | 0.160 | 0.0520 | 0.082 | 0.289 |  | 0.087 | 0.0312 | 0.042 | | 0.170 |
| pent4 | 0.127 | 0.0448 | 0.062 | 0.243 |  | 0.087 | 0.0312 | 0.042 | | 0.170 |
|  |  |  |  |  |  |  |  |  | |  |
|  | | |  |  |  | . | | |  |  |

*Three models included. *Six models included

Only pent(·) models were included.

| 3A) Pihlajavesi, years 2013-17 | | | | |
| --- | --- | --- | --- | --- |
|  | Combined, N obs 115 | | |  |
|  |  |  | 95% C. I. | |
| Parameter | Estimate* | SE | Lower | Upper |
| Phi1 | 0.888 | 0.0334 | 0.805 | 0.939 |
| Phi2 | 0.887 | 0.0326 | 0.806 | 0.937 |
| Phi3 | 0.891 | 0.0357 | 0.799 | 0.944 |
| Phi4 | 0.874 | 0.0635 | 0.691 | 0.955 |
| p1 | 0.764 | 0.1116 | 0.491 | 0.916 |
| p2 | 0.710 | 0.0571 | 0.587 | 0.808 |
| p3 | 0.724 | 0.0486 | 0.619 | 0.808 |
| p4 | 0.743 | 0.0590 | 0.612 | 0.842 |
| p5 | 0.716 | 0.0681 | 0.567 | 0.830 |
| pent1 | 0.172 | 0.0744 | 0.069 | 0.366 |
| pent2 | 0.084 | 0.0521 | 0.024 | 0.257 |
| pent3 | 0.218 | 0.0704 | 0.110 | 0.385 |
| pent4 | 0.088 | 0.0488 | 0.029 | 0.241 |
|  |  |  |  |  |

*All eight models included

| 1B) Haukivesi, years 2010-12 | | | |  |  | 2B) Haukivesi, years 2010-17 | | | | |
| --- | --- | --- | --- | --- | --- | --- | --- | --- | --- | --- |
| Camera trap, N obs 44 | | |  |  |  | Boat survey, N obs 68 | | |  | |
|  |  |  | 95% C. I. | |  |  |  | 95% C. I. |  | |
| Parameter | Estimate* | SE | Lower | Upper |  | Estimate* | SE | Lower | Upper | |
| Phi1 | 0.813 | 0.1168 | 0.491 | 0.951 |  | 0.929 | 0.0220 | 0.872 | 0.961 | |
| Phi2 | 0.805 | 0.1156 | 0.494 | 0.946 |  | 0.929 | 0.0220 | 0.872 | 0.961 | |
| Phi3 |  |  |  |  |  | 0.929 | 0.0220 | 0.872 | 0.961 | |
| Phi4 |  |  |  |  |  | 0.929 | 0.0220 | 0.872 | 0.961 | |
| Phi5 |  |  |  |  |  | 0.929 | 0.0220 | 0.872 | 0.961 | |
| Phi6 |  |  |  |  |  | 0.929 | 0.0220 | 0.872 | 0.961 | |
| Phi7 |  |  |  |  |  | 0.929 | 0.0220 | 0.872 | 0.961 | |
| p1 | 0.811 | 0.2128 | 0.220 | 0.985 |  | 0.508 | 0.1313 | 0.269 | 0.743 | |
| p2 | 0.680 | 0.1314 | 0.394 | 0.874 |  | 0.378 | 0.0947 | 0.216 | 0.572 | |
| p3 | 0.850 | 0.1914 | 0.230 | 0.991 |  | 0.631 | 0.0929 | 0.438 | 0.789 | |
| p4 |  |  |  |  |  | 0.469 | 0.0808 | 0.318 | 0.625 | |
| p5 |  |  |  |  |  | 0.362 | 0.0757 | 0.230 | 0.519 | |
| p6 |  |  |  |  |  | 0.744 | 0.0728 | 0.578 | 0.860 | |
| p7 |  |  |  |  |  | 0.750 | 0.0789 | 0.568 | 0.873 | |
| p8 |  |  |  |  |  | 0.647 | 0.0851 | 0.469 | 0.792 | |
| pent1 | 0.217 | 0.1240 | 0.062 | 0.537 |  | 0.079 | 0.0147 | 0.054 | 0.113 | |
| pent2 | 0.183 | 0.0881 | 0.066 | 0.415 |  | 0.079 | 0.0147 | 0.054 | 0.113 | |
| pent3 |  |  |  |  |  | 0.079 | 0.0147 | 0.054 | 0.113 | |
| pent4 |  |  |  |  |  | 0.079 | 0.0147 | 0.054 | 0.113 | |
| pent5 |  |  |  |  |  | 0.079 | 0.0147 | 0.054 | 0.113 | |
| pent6 |  |  |  |  |  | 0.079 | 0.0147 | 0.054 | 0.113 | |
| pent7 |  |  |  |  |  | 0.079 | 0.0147 | 0.054 | 0.113 | |
|  |  |  |  |  |  |  |  |  |  | |

*Four models included *Three models included: due to Akaike's

weight's only the Phi.dot.p.time.pent.dot -

model has practical effect on parameters
